# JUN is important for ocular hypertension-induced retinal ganglion cell degeneration

**DOI:** 10.1038/cddis.2017.338

**Published:** 2017-07-20

**Authors:** Stephanie B Syc-Mazurek, Kimberly A Fernandes, Richard T Libby

**Affiliations:** 1 Flaum Eye Institute, University of Rochester Medical Center, Rochester, NY 14642, USA; 2 Neuroscience Graduate Program, University of Rochester Medical Center, Rochester, NY 14642, USA; 3 Department of Biomedical Genetics, University of Rochester Medical Center, Rochester, NY 14642, USA; 4 The Center for Visual Sciences, University of Rochester Medical Center, Rochester, NY 14642, USA

## Abstract

Ocular hypertension, a major risk factor for glaucoma, is thought to trigger glaucomatous neurodegeneration through injury to retinal ganglion cell (RGC) axons. The molecular signaling pathway leading from ocular hypertension to RGC degeneration, however, is not well defined. JNK signaling, a component of the mitogen-activated protein kinase (MAPK) family, and its canonical target, the transcription factor JUN, have been shown to regulate neurodegeneration in many different systems. JUN is expressed after glaucoma-relevant injuries and *Jun* deficiency protects RGCs after mechanical injury to the optic nerve. Here, we tested the importance of JNK–JUN signaling for RGC death after ocular hypertensive axonal injury in an age-related, mouse model of ocular hypertension. Immunohistochemistry was performed to evaluate JUN expression in ocular hypertensive DBA/2J mice. JUN was expressed in a temporal and spatial pattern consistent with a role in glaucomatous injury. To determine the importance of JUN in ocular hypertension-induced RGC death, a floxed allele of *Jun* and a retinal expressed cre recombinase (Six3-cre) were backcrossed onto the DBA/2J background. Intraocular pressure (IOP) and gross morphology of the retina and optic nerve head were assessed to determine whether removing *Jun* from the developing retina altered IOP elevation or retinal development. *Jun* deficiency in the retina did not alter DBA/2J IOP elevation or retinal development. Optic nerves and retinas were assessed at ages known to have glaucomatous damage in DBA/2J mice. *Jun* deficiency protected RGC somas from ocular hypertensive injury, but did not protect RGC axons from glaucomatous neurodegeneration. *Jun* is a major regulator of RGC somal degeneration after glaucomatous ocular hypertensive injury. These results suggest in glaucomatous neurodegeneration, JNK–JUN signaling has a major role as a pro-death signaling pathway between axonal injury and somal degeneration.

Glaucoma is characterized by stereotypical death of retinal ganglion cells (RGCs). Ocular hypertension, a major risk factor for glaucoma, causes glaucomatous neurodegeneration by injuring RGC axons.^1, 2^ Ocular hypertension-induced axonal injury is thought to occur to RGCs as they exit through a specialized structure, the lamina cribrosa.^3, 4, 5, 6, 7^ Within RGCs, axonal injury triggers a molecular cascade that ultimately leads to the apoptotic death of RGCs.^8^ Activation of BAX, a molecule important for triggering the final events in the apoptotic cascade, is known to be critical for axonal injury induced RGC death.^9, 10, 11, 12^ Unfortunately, however, the vast majority of the molecular pathway leading from axonal injury to RGC apoptosis in glaucoma remains unclear.

After axonal injury, phosphorylation-dependent signals are among the first changes to occur within the axon.^13, 14^ These signaling pathways are known to alter a cell’s transcriptional response to injury as well as control its viability. Glaucoma-relevant insults, such as cytoskeleton disruption, neurotropic deprivation, and extrinsic pro-inflammatory signals all can activate phosphorylation-dependent signaling cascades. The mitogen-activated protein kinase (MAPK) family is a phosphorylation-dependent signaling system known to be an important pro-death signaling pathway in injured neurons, including after glaucoma-relevant injuries.^15, 16^ In particular, c-Jun *N*-terminal kinases (JNKs), members of the MAPK family, have been suggested to be important in neurodegeneration.^15, 16^ JNK signaling is activated in RGCs after glaucoma-relevant injuries and pJNK is present in RGCs in human glaucoma patients.^13, 17, 18, 19, 20, 21, 22, 23, 24, 25, 26^ Several studies have shown that inhibiting JNK activation lessens/delays RGC death after glaucoma-relevant insults.^18, 19, 27^ Thus, multiple lines of evidence support JNK signaling being involved in glaucomatous neurodegeneration.

The canonical target of pathological JNK signaling is the transcription factor JUN. JUN is an AP-1 family transcription factor. AP-1 family transcriptional activity is known to be an early critical component of the neuronal axonal injury response.^28^ JUN is activated in RGCs after several glaucoma-relevant insults such as excitotoxicity, optic nerve injury, and elevated intraocular pressure (IOP).^18, 19, 20, 21, 22^ We recently showed that RGC death after mechanical axonal injury is significantly reduced in mice with retinal-specific deletion of *Jun*.^19^ In fact, *Jun* deficiency prevented RGC death during the first two weeks after controlled optic nerve crush (CONC), a result similar to that observed in *Bax*-deficient mice, though *Jun* deficiency did not provide the complete long term protection of *Bax* deficiency.^9, 12, 19, 29^ These data support the hypothesis that JUN activation is critical for RGC death in glaucoma, similar to acute models of axon injury. However, because RGC death in glaucoma may differ from experimentally induced axonal injury – in the magnitude of insult, kinetics of RGC cell loss, or extrinsic triggers (glial signaling) *versus* intrinsic triggers (cytoskeleton disruption) – the role of JUN and JNK must be tested in an ocular hypertensive glaucoma model.^30, 31^ Furthermore, increasing evidence points to transcriptional events being key mediators of neuronal degeneration. In some instances, transcriptional changes are required for axonal degeneration or, alternatively, for regenerative events and increasing axonal stability after injury.^32, 33, 34^ Here, we directly test the importance of JUN in regulating axonal and somal degeneration in an age-related, stochastic model of ocular hypertension, the DBA/2J mouse. We find that JUN is expressed in this model in a temporal and spatial pattern consistent with a role in glaucomatous neurodegeneration. Though *Jun* does not appear to have a role in axonal degeneration after ocular hypertensive injury, *Jun* was found to be a major regulator of RGC somal degeneration after ocular hypertensive injury. These results suggest that JNK–JUN signaling has a major role as a pro-death signaling pathway between axonal injury and somal degeneration in glaucomatous neurodegeneration.

## Results

### JUN is expressed in aged DBA/2J mice

**Figure 1:**
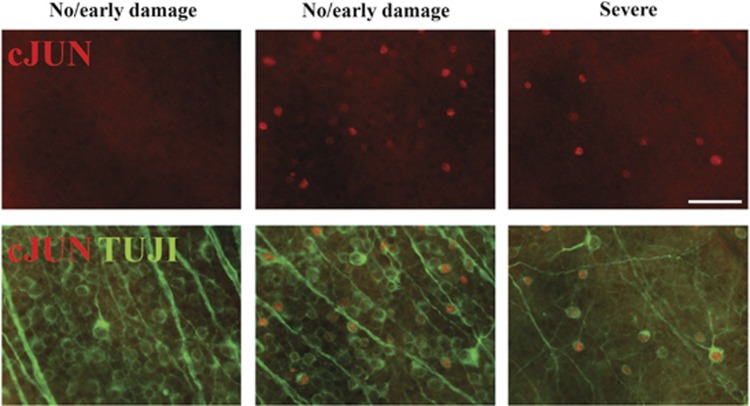
JUN is expressed in ocular hypertensive mice. To determine whether JUN expression was consistent with a role in ocular hypertension-induced RGC death, retinal flat mounts (RGC cell up) were examined from 10.5-month-old DBA/2J mice. Retinas with corresponding optic nerves with no (no/early nerves) or severe glaucomatous damage were assessed. JUN (red) accumulates in RGCs (labeled with TUJI, a marker of RGCs, green) in the retina of nerves with both no/early and severe damage at 10.5 months of age. Two representative images from the same retina with a corresponding no/early damage nerve are shown to demonstrate the heterogeneity of JUN expression. JUN was expressed in 8 of 10 eyes evaluated. JUN (red) is expressed in a temporal and spatial pattern consistent with a function in ocular hypertensive-induced RGC death. Scale bar: 50 *μ*m

The DBA/2J mouse is an age-related, stochastic model of ocular hypertension.^35, 36^ Mutations in two DBA/2J genes, *Gpnmb* and *Tyrp1,* cause an iris disease that leads to age-related ocular hypertension.^37^ The increased IOP results in significant RGC death and axonal degeneration in the majority of DBA/2J eyes.^38^ Genetic, surgical, and pharmacologic studies have demonstrated that DBA/2J glaucomatous phenotypes are a result of ocular hypertension.^39, 40, 41, 42^ In DBA/2J mice, IOP increases from 6–12 months of age, and the majority of DBA/2J mice demonstrate severe glaucomatous damage by 12 months of age.^35^ To determine whether JUN signaling is active in RGCs after ocular hypertensive insult, the expression of JUN was evaluated in 10.5 month DBA/2J mice, a time point when approximately half of all DBA/2J retinas have clear, morphological signs of glaucomatous damage.^35^ JUN expression was upregulated in 80% of retinas evaluated (*n*=10) consistent with the asynchronous onset of ocular hypertension (Figure 1). JUN accumulated in RGCs in the retina of nerves with no/early and severe glaucomatous damage (see methods). JUN was expressed in patches in retinas with corresponding nerves with no/early damage and was widely expressed in retinas with corresponding moderate or severe nerves. Thus, JUN was expressed in a temporal and spatial pattern consistent with this transcription factor functioning during the window of RGC death in DBA/2J ocular hypertension.

### Establishing and validating *Jun*-deficient DBA/2J mice

**Figure 2:**
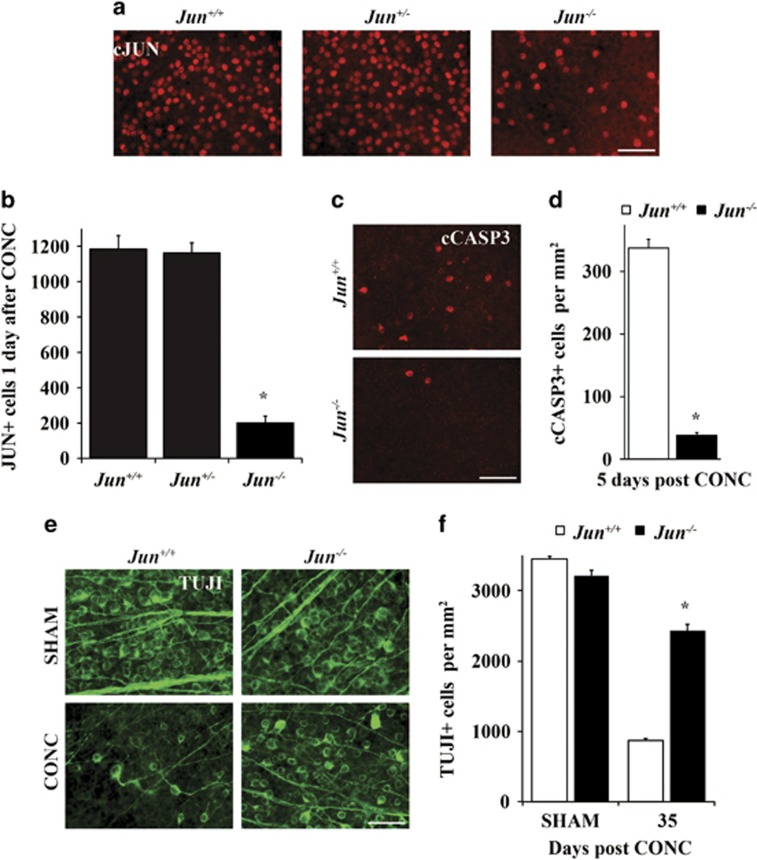
Establishing and validating *Jun*-deficient DBA/2J mice. (**a**) To determine the recombination efficiency of Six3-cre, the number of JUN+ cells were counted in retinal flat mounts 1 day after controlled optic nerve crush (CONC), a time prior to RGC death when JUN is widely expressed. (**b**) JUN+ cells were reduced by 83% in *Jun*-deficient retinas (error bars represent S.E.M.; *n*=6 per genotype; *P*<0.001; scale bar: 50 *μ*m). To ensure genetic background did not alter the protection afforded by *Jun* deficiency for RGC death after axonal injury, CONC was performed in young DBA/2J animals (<5 months of age). (**c, d**) Similar to the protection from CONC observed on the C57BL/6J background,^18^ *Jun*-deficient animals had 88.7% fewer cleaved caspase 3+cells (cCASP3, red) as compared with control animals (*P*<0.001, *n*=6). (**e, f**) *Jun-*deficient animals had significantly greater RGC survival (TUJ1+ cells, green) than wild-type animals 35 days after CONC, a time point when the majority of RGCs will have died after CONC (WT: 25.1% survival, *Jun*-deficient: 75.5% survival; *, *P*<0.001, *n*=6; scale bars: 50 *μ*m). Error bars represent S.E.M.

As JUN was expressed after ocular hypertensive injury, a floxed allele of *Jun* and Six3-cre (a neural retina cre) were backcrossed into the DBA/2J mouse to test the importance of JUN signaling in glaucomatous neurodegeneration.^43, 44^ As recombination efficiency can differ between mouse strains and cell type, the recombination efficiency of Six3-cre was assessed in the DBA/2J genetic background. The number of JUN+ cells were counted in retinal flat mounts 1 day after CONC, a time point prior to RGC death when JUN is widely expressed in injured RGCs.^19^ JUN+ cells were reduced in *Jun*-deficient retinas as compared with wild-type retinas and *Jun* heterozygous retinas by 83.1 and 82.7%, respectively (Figures 2a and b, *P*<0.001, *n*=6). No significant difference was observed in JUN expression between *Jun* wild-type retinas and *Jun* heterozygous retinas. Thus, Six3-cre appears to provide complete recombination of *Jun* from over 80% of RGCs on the DBA/2J genetic background.

*Jun* deficiency has been shown previously to protect RGCs after mechanical optic nerve insult in C57BL/6J animals.^19^ Different genetic backgrounds can affect RGC death after axonal insult.^12, 45, 46^ To ensure the DBA/2J genetic background did not alter the protection afforded by *Jun* deficiency for RGC death after axonal injury, CONC was performed in young DBA/2J animals (<5 months of age), prior to the development of increased IOP.^35^ Similar to the protection observed on the C57BL/6J background,^18^ *Jun*-deficient animals had 88.7% fewer dying RGCs (stained with cleaved caspase) as compared with wild-type animals 5 days after axonal injury (Figures 2 c and d, *P*<0.001, *n*=6). In addition, 35 days after CONC *Jun-*deficient animals had greater RGC survival as compared with wild-type controls (Figures 2 e and f, 75.5% survival in *Jun-*deficient *versus* 25.1% survival in WT animals, *P*<0.001, *n*=6). Thus, *Jun* deficiency conferred similar protection to RGCs after acute axonal injury in both C57BL/6J and DBA/2J mice.

### *Jun* deficiency does not alter the glaucoma-relevant endo-phenotypes of DBA/2J mice

**Figure 3:**
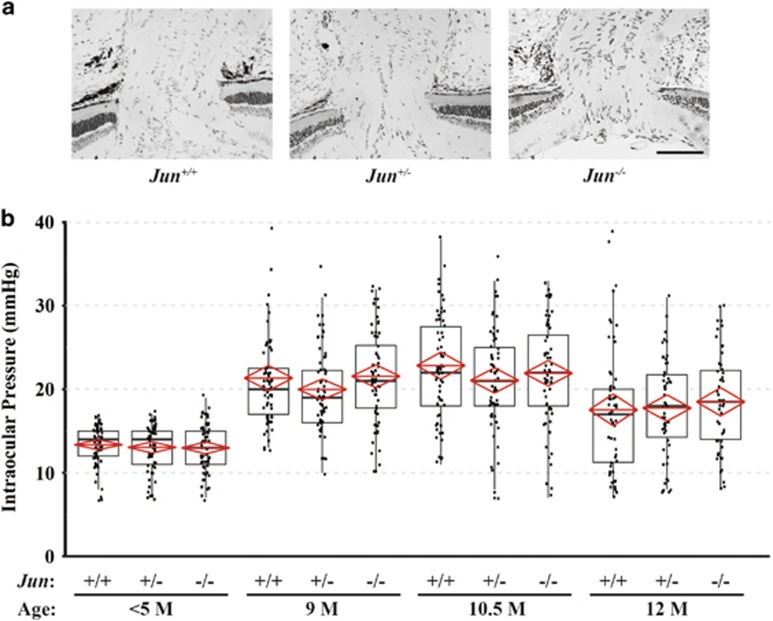
*Jun* deficiency does not alter the glaucoma-relevant endo-phenotypes of DBA/2J mice. (**a**) Semi-thin retinal cross sections were taken through the optic nerve head so that gross structure of the optic nerve head could be examined in *Jun*-deficient animals prior to the onset of IOP elevation (<5 months of age). *Jun* deficiency did not appear to alter the gross morphology of these structures (scale bar 100 *μ*m). (**b**) To ensure *Jun* deficiency did not alter the intraocular pressure (IOP) profile of DBA/2J mice, IOPs were measured at 3, 9, 10.5, and 12 months (Tonolab, Icare). No significant difference was seen between *Jun*-deficient and wild-type mice at any time point (*P*>0.05 for all comparisons; *n*=64 per genotype for <5M, 9M, 10.5M; *n*≥48 per genotype for 12M). Note, in all three genotypes, IOP was significantly elevated at 9, 10.5, and 12 months of age compared to <5M mice (*P*<0.001 for all comparisons). Solid black line represents the median while the lower and upper boundaries of the black box represent the 75th and 25th percentile, respectively. The red line represents the mean and the top and bottom points of the red diamond represent the 95% confidence interval

The optic nerve head is a critical structure in the primary pathophysiology of glaucoma and thus altering the morphology of this structure could influence glaucomatous phenotypes.^3, 7^ Genetic manipulation of a known JUN target gene, *Bim*, has been shown to alter vascular and optic nerve head morphology in DBA/2J mice.^47, 48^ To determine whether *Jun* deficiency altered optic nerve head morphology, *Jun*-deficient, *Jun* heterozygous, and wild-type animals were evaluated prior to the development of IOP elevation (<5 months). *Jun* deficiency did not appear to alter gross optic nerve head or retinal morphology (Figure 3a and data not shown).

Glaucomatous neurodegeneration in DBA/2J mice is dependent on IOP elevation.^39, 40, 41, 42^ To ensure *Jun* deficiency did not alter the IOP profile of DBA/2J mice, IOPs of *Jun*-deficient, *Jun* heterozygous, and wild-type animals were measured at a time point before IOP is known to become elevated (3–5 months of age). Not surprisingly, as *Jun* was not deleted from the ocular structures controlling IOP regulation, *Jun* deficiency did not alter IOP levels in young mice (Figure 3b). IOPs were also measured at time points where DBA/2J mice are known to be ocular hypertensive (9, 10.5, and 12 months of age). IOP measurements at 9M, 10.5M, and 12M of age were significantly increased compared with animals <5 months of age for all genotypes (*P*<0.05). Furthermore, there were no significant differences observed at any time point across the different genotypes (Figure 3b, *n*=64 per genotype for <5M, 9M, 10.5M; *n*≥48 per genotype for 12M; *P*>0.05 for all comparisons within a time point). Together these data suggest that *Jun* deficiency does not alter glaucoma-relevant endo-phenotypes such as optic nerve morphology and IOP profile in DBA/2J mice.

### *Jun* deficiency does not prevent ocular hypertension-induced optic nerve degeneration

**Figure 4:**
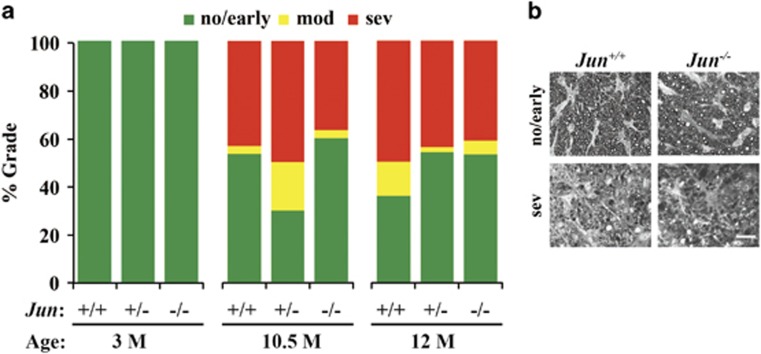
*Jun* deficiency does not prevent ocular hypertension-induced optic nerve degeneration. To assess the influence of *Jun* deficiency on ocular hypertension-induced axonal degeneration, optic nerves from DBA/2J mice at 3, 10.5, and 12 months of age were stained and graded on a scale measuring optic nerve damage (no/early, moderate (mod) or severe (sev)). (**a**) *Jun* deficiency did not prevent ocular hypertension-induced optic nerve degeneration. In fact, *Jun* deficiency did not lesson optic nerve damage at either 10.5 or 12 months of age (3M: *n*=10 per genotype; 10.5M *n*=30 per genotype; 12M: *n*≥ 50 per genotype). (**b**) Examples of no/early and severe nerves for WT and *Jun-*deficient mice are shown. Scale bar: 20 *μ*m

To assess the influence of *Jun* deficiency on DBA/2J ocular hypertension-induced axonal degeneration, optic nerves from mice at various ages were stained with paraphenylenediamine (PPD) and graded on a scale measuring glaucomatous optic nerve damage (no/early, moderate, or severe depending on axonal loss and gliosis; see methods for more details about the scoring criteria).^4, 9, 47^ Prior to IOP elevation (mice between 1.5–5 months of age) no glaucomatous damage was observed in any genotype (Figure 4; *n*=10 for each genotype; *P*>0.05 for each comparison). At 12 months of age, a time point when most of the axonal damage that will happen has already occurred in DBA/2J mice,^35^ *Jun* deficiency did not lessen axonal damage (Figure 4; *n*≥50 for each genotype; *P*>0.05 for all comparisons). By using DBA/2J mice at an earlier time point (10.5 months), several studies have shown that either genetic or pharmaceutical manipulation can delay glaucomatous damage.^9, 49, 50^ Therefore, a cohort of DBA/2J mice was examined at 10.5 months of age (*n*=30 for all genotypes). At 10.5 months, ~50% of nerves demonstrate severe optic nerve pathology in the wild-type animals. Similar to 12 months of age, there was no significant difference in optic nerve damage level between any genotype (Figure 4; *P*>0.05 for all comparisons). *Jun* deficiency therefore does not appear to prevent or delay ocular hypertension-induced axonal damage in DBA/2J mice.

### JUN is important for ocular hypertension-induced RGC degeneration

**Figure 5:**
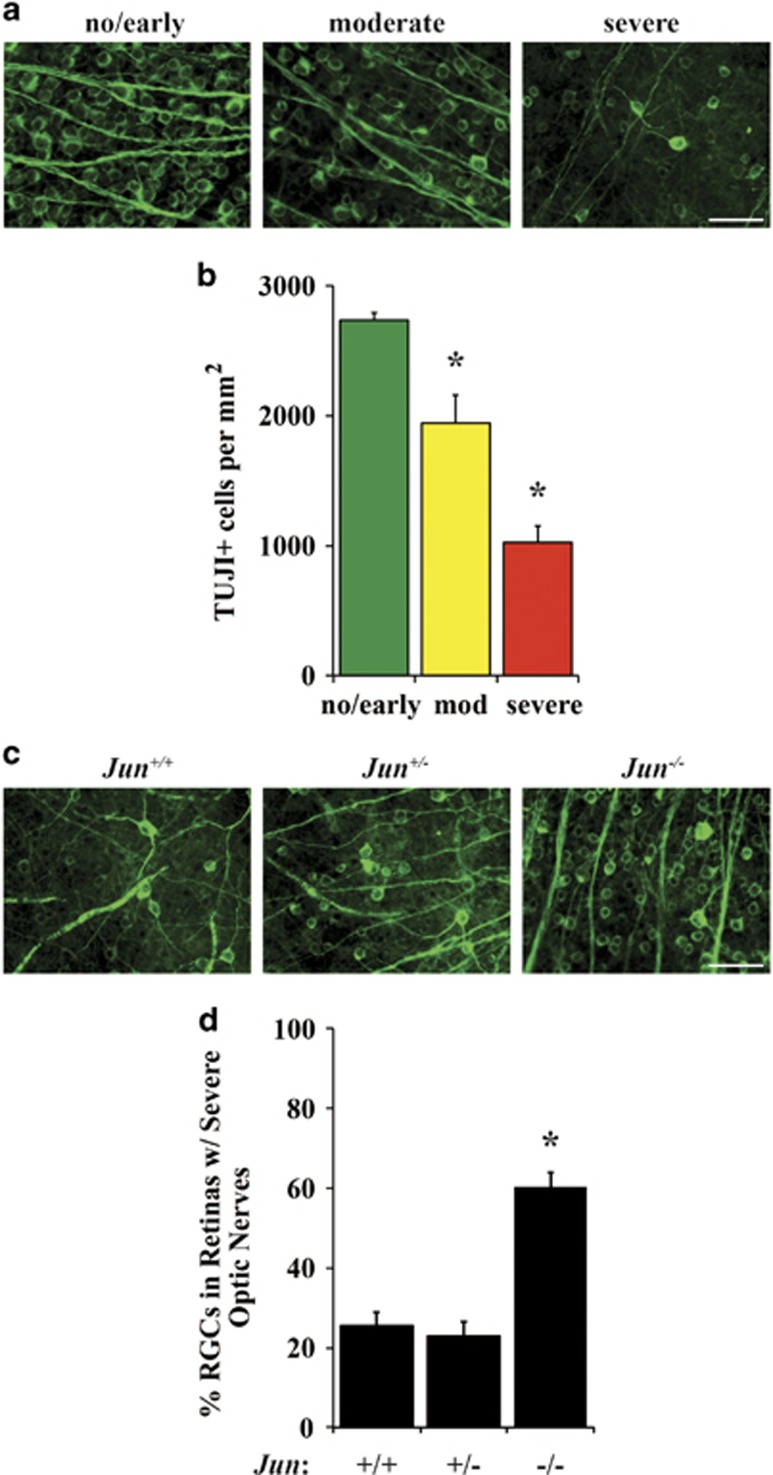
JUN is important for ocular hypertension-induced RGC degeneration. RGCs were counted in wild-type DBA/2J mice to determine the relationship between RGC survival and optic nerve severity (**a**; stained with TUJI). (**b**) Quantification of RGCs demonstrated increased optic nerve damage was associated with decreased RGC survival (*n*≥8 per group, *P*<0.05). To determine whether *Jun* deficiency protected RGC somas after ocular hypertension-induced injury, RGCs (**c**); stained with TUJI) were counted in retinal flat mounts with corresponding nerves with severe optic nerve damage (nerves judged to have<5% of axons surviving). (**d**) Quantification of *Jun* wild-type and *Jun*-deficient eyes with severely degenerated optic nerves showed that *Jun* deficiency provided significant protection to RGC somas compared to WT eyes; data normalized to young (<5 month) control animals (WT: 25.5% survival, *Jun*-deficient: 60.4% survival; *, *P*<0.001, *n*≥12; scale bars 50 *μ*m)

After ocular hypertensive injury, separate molecular mechanisms are known to contribute to RGC axonal and somal degeneration.^4, 9, 30, 51, 52, 53, 54^ For instance, *Bax* deficiency protected RGC somas, but not axons from ocular hypertension-induced RGC death.^9^ Furthermore, after mechanical axonal injury, we have shown that deficiency of *Dlk* or *Jnk2* and *Jnk3* protects RGCs from somal degeneration, but not axonal degeneration.^18, 55^ Therefore, it is possible that *Jun* deficiency could have an important role in somal loss in ocular hypertensive DBA/2J mice. To establish the relationship between RGC survival and optic nerve severity, RGCs were counted in aged wild-type DBA/2J mice. Increased optic nerve damage was associated with decreased RGC survival (Figures 5a and b; *n*≥8 per group, *P*<0.05). To determine whether *Jun* deficiency protected RGCs somas, RGCs were counted in retinal flat mounts from 10.5 and 12-month-old eyes with corresponding nerves that were judged to have massive axonal loss (nerves judged to have <5% of axons surviving). Wild-type and heterozygous animals had similar amounts of RGC somal loss, as judged by TUJ1+ cells (25.5% and 23.0%, respectively; Figures 5 c and d). *Jun* deficiency provided significant protection to 60.4% of RGCs with severe optic nerve damage as compared with wild-type and heterozygous mice (Figures 5c and d; *n*≥12 per genotype, *P*<0.05). The RGC somal protection conferred by *Jun* deficiency may be even higher as ~17% of RGCs still express JUN (due to incomplete recombination of the *Jun* allele by Six3-cre), suggesting that perhaps ~75% of RGCs may survive in animals with massive glaucomatous damage to their optic nerves. These data demonstrate an important role for *Jun* in ocular hypertension-induced RGC death.

## Discussion

Ocular hypertension, a leading risk factor for glaucoma, injures RGC axons and kills RGCs.^1, 3, 7, 56^ Distinct molecular signaling pathways have been shown to govern somal and axonal degeneration after glaucomatous ocular hypertension injury.^4, 9, 30, 51, 52, 53, 54^ The specific signaling cascade(s) leading to RGC somal and axonal degeneration after a glaucomatous injury is not known. As glaucomatous damage is likely due to axonal injury, models of axonal injury have been used to study the molecular signaling pathways critical for glaucomatous neurodegeneration. Mechanical optic nerve injury (optic nerve crush and optic nerve transection) provides an acute model for investigation of axonal injury-induced RGC death. There are, however, important differences between mechanical optic nerve injury and ocular hypertension. For instance, the expression of the Wallerian degeneration slow mutation (*Wld*^*S*^) significantly slows RGC axonal and somal degeneration in glaucoma, but does not lessen RGC apoptosis after optic nerve crush.^4, 53, 54, 57^ Furthermore, multiple studies have suggested glia and blood derived cells are involved in ocular hypertension-induced RGC death; events that may not be required after direct axonal injury.^58, 59, 60, 61, 62, 63^ Thus, to determine the critical molecular signaling events that govern both axonal and somal glaucomatous neurodegeneration, it is necessary to test molecules in a model of ocular hypertension. In the present work, the role of *Jun,* a member of the MAPK signaling family, which has been implicated in many glaucoma-relevant injuries, was critically tested for its importance in ocular hypertension-induced RGC death. In ocular hypertensive DBA/2J mice, JUN was expressed prior to RGC death after ocular hypertensive injury. Furthermore, *Jun* deficiency protected RGC somas, but not axons from ocular hypertension-induced glaucomatous neurodegeneration.

*Jun* signaling is important for RGC somal degeneration in ocular hypertensive DBA/2J mice. *Jun* deficiency significantly protected RGC somas in eyes with severe glaucomatous axonal injury. Ultimately, as BAX activation has been shown to be required for RGC death in DBA/2J mice, a JUN-dependent pro-death signaling pathway must converge on BAX activation.^8, 9, 12^ Since JUN is a transcription factor, it is likely that JUN controls the expression of genes that contribute to BAX activation. In RGCs JUN has been shown to control several molecules involved in RGC death after axonal injury.^19, 47^ For instance, *Bim*, a member of the pro-death Bcl-2 family of proteins known to activate BAX, is regulated by JUN in RGCs. *Bim* deficiency also lessens RGC death after mechanical axonal injury.^47^ However, *Bim* deficiency did not lessen RGC somal degeneration in ocular hypertensive mice. These data suggest other pro-death Bcl-2 family members are directly or indirectly controlled by JUN activation and contribute to ocular hypertension-induced RGC death. *Atf3,* which like *Jun* is also a member of the AP-1 family of transcription factors, has been suggested to be involved in glaucomatous neurodegeneration.^64, 65, 66, 67^ ATF3 expression is controlled by JUN in RGCs, however, deficiency in *Atf3* only provides minor protection to RGCs after mechanical axonal injury compared with *Jun* deficiency.^19^ Therefore, the key downstream *Jun*-dependent pro-death transcriptional events linking JUN activation to BAX activation in ocular hypertensive eyes are undefined. Furthermore, in addition to the pro-death signaling role of JUN, JUN has been shown to regulate pro-survival signaling.^19, 68, 69, 70^ A pro-survival role for JUN may account for the lost RGCs in *Jun*-deficient mice and/or the axonal degeneration in ocular hypertensive DBA/2J mice. JUN could be a major transcriptional hub in RGCs, controlling pro-survival, pro-death and pro-regenerative pathways.^19^ Detailed study of the downstream targets of JUN, including determining whether JUN targets change with the duration or magnitude of an ocular hypertensive insult is an important next step in understanding JUN’s role in injured RGCs. Understanding the gene network controlled by JUN may lead to a better understanding of how RGCs respond to an ocular hypertensive injury and how ultimately, they either die or survive that insult.

In addition to identifying downstream transcriptional targets, determining the signaling molecules upstream of *Jun* is an important step in understanding the molecular degeneration cascade triggered by an ocular hypertensive injury. Several members of the MAPK signaling family upstream of *Jun*, including *Jnk2*, *Jnk3*, and *Dlk* have been found to be important for RGC death after axonal injury.^18, 55, 71, 72^ Identifying the upstream targets of *Jun* may also help uncover the inciting insult leading to RGC death and axonal degeneration. Further defining the molecular role of the MAPK family, including potential non-canonical roles for MAPK signaling, in addition to evaluating non-MAPK family members will be necessary to define the signaling cascade leading to RGC death in ocular hypertension.

Although *Jun* deficiency protects RGCs somas after ocular hypertension injury, *Jun* is not required for axonal degeneration. Dual leucine kinase (DLK), a mitogen-activated protein kinase kinase kinase (MAP3K) upstream of JNK/JUN signaling, is also an important regulator of RGC death after axonal injury.^55, 71, 72^ Furthermore, *Dlk* has been implicated in axonal degeneration after ocular hypertension injury. Pharmacological inhibition using a kinase inhibitor (Tozasertib; a non specific inhibitor of DLK^73, 74^) was reported to protect RGCs and optic nerve axons in an acute, ocular hypertensive model of glaucoma.^72^ In addition to *Dlk* deficiency providing RGC protection and moderate axonal protection after acute axonal injury, DLK has been shown to have a role in axonal degeneration in other models. *Dlk* deficiency delays axonal degeneration after sciatic nerve transection and axotomy of dorsal root ganglia.^75^ Downstream of DLK, pharmacologic inhibition of JNK signaling delays axonal degeneration after axonal injury in drosophila and cultured mammalian neurons.^76^ Despite the role of MAPK signaling in ocular hypertension-induced axonal degeneration as evidenced by the involvement of DLK and JNK, axonal degeneration after ocular hypertension must proceed through non-canonical DLK signaling because deficiency of its canonical downstream effector, *Jun*, does not prevent axonal degeneration. Furthermore, pharmacologic inhibition of DLK did not provide complete protection to the axonal compartment after ocular hypertension and *Dlk* deficiency did not prevent axonal degeneration after CONC.^55, 72^ Together, these results suggest another pro-death signaling pathway contributes to RGC axonal degeneration. Further study of other signaling molecules suggested to have a role in axonal degeneration such as DDIT3, IKK, GSK3, or PHR1 will be necessary to understand the signaling pathways for axonal degeneration after ocular hypertension.^77, 78, 79^

## Conclusion

JUN was expressed in RGCs in a spatial and temporal pattern consistent with a role in RGC death after ocular hypertensive injury, an important risk factor for glaucoma. *Jun* deficiency did not provide protection to RGC axons in the ocular hypertensive injury but did provide robust protection to RGC somas. It is important to note *Jun* deficiency did not provide complete protection to RGCs even after accounting for incomplete recombination of the *Jun* allele. Consequently, activation of another pro-death signaling molecule upstream of *Bax* must be crucial for ocular hypertension-induced RGC somal death. JUN has both pro-death and pro-survival roles in RGCs after axonal injury to RGCs, making it possible that JUN-dependent pro-survival signaling is important for maintaining RGC viability after an ocular hypertensive injury. JUN appears to have an important role in RGCs after axonal injury, likely acting as a transcriptional hub controlling molecular pathways integral to the viability of RGCs. It will be important to understand the transcriptional network JUN controls as well as determine whether these networks change with the duration or intensity of ocular hypertension. Furthermore, it will be important to understand the molecular pathway that leads to JUN activation after a glaucomatous insult. Such study is likely to lead to the initiating events in RGCs that trigger axonal injury-induced RGC death. JUN has an important role in the death of RGCs after an ocular hypertensive insult.

## Materials and methods

### Mice

Mice were fed chow and water ad libitum and housed on a 12 h light to dark cycle. All experiments were conducted in adherence with the Association for Research in Vision and Ophthalmology's statement on the use of animals in ophthalmic and vision research and were approved by the University of Rochester's University Committee on Animal Resources. A floxed allele of *Jun*^43^ and Six3-cre recombinase (a neural retina cre)^44^ were backcrossed onto the DBA/2J background for at least seven generations. During the backcrossing, mice were genotyped to ensure the colony was homozygous for the *Gpnmb* and *Tyrp1* alleles that impart iris disease and secondary IOP elevation in DBA/2J mice.^37, 80^ Following the backcross, animals were intercrossed to generate animals (1) carrying the recombined floxed alleles, referred to as *Jun*^−^^/−^ or *Jun*-deficient (*Jun*^*fl/fl*^Six3cre^+^) (2) heterozygote animals referred to as *Jun*^+/^^−^(*Jun*^*+*/fl^Six3cre^+^), and (3) animals carrying non-recombined floxed alleles or wild-type alleles with or without the cre recombinase referred to as *Jun*^+/+^ or wild-type (WT; *Jun*^*+/+*^Six3*cre*^*−*^, *Jun*^*+/+*^Six3*cre*^*+*^, *Jun*^*+/fl*^Six3*cre*^*−*^, or *Jun*^*fl/fl*^ Six3*cre*^*−*^). Since there is a known small difference in IOP elevation and nerve damaged levels between male and female DBA/2J mice,^35^ approximately equal numbers of male and female animals were used for the experiments assessing glaucomatous damage (between 47 and 53% for each sex for each condition and time point).

### Mechanical optic nerve injury and glaucoma

CONC was performed on young DBA/2J mice anesthetized with 100 mg/kg ketamine and 10 mg/kg xylazine as previously described.^9, 18, 19, 29, 47, 55^ In brief, the optic nerve was exposed and then crushed with self-closing forceps for 5 s immediately behind the eye. Sham surgery was performed in control eyes where the optic nerve was exposed but not crushed. Animal cohorts were harvested 1, 5, and 35 days following CONC. IOPs were measured using the TonoLab (Colonial Medical Supply, Franconia, NH, USA) at 3–5, 9, 10.5, and 12 months of age. IOPs were recorded according to manufacturer’s instructions three to five minutes after administration of anesthetic 100 mg/kg ketamine and 10 mg/kg xylazine. To determine the level of glaucomatous nerve damage, nerves were stained with PPD as has been previously described.^4, 9, 47^ In brief, nerves were dissected, processed, embedded in Technovit 7100 (Electron Microscopy Sciences, Hatfield, PA, USA), cut (1.5 *μ*m sections), and stained with PPD. PPD stains the myelin sheath of all axons but differentially darkly stains the axoplasm of dying axons. A validated grading scale was used to assess the level of glaucomatous damage as has been previously described:^4, 9, 47^ no or early nerves were judged to have <5% of the axons lost or damaged, a number that is consistent with age-related damage; moderate nerves were judged to have many damaged axons throughout the nerve averaging ~30% of the axons damaged or lost often with localized signs of gliosis; severe nerves were judged to have >50% of the axons lost or damaged and often with large areas of glial scarring.

### Retinal histology and cell counts

For plastic sectioning, eyes were fixed for 24 h in a solution of 2.5% gluteraldehyde, 2% paraformaldehyde (PFA) at 4 °C. Eyes were then dehydrated, embedded in Technovit 7100 and sectioned at 2.5* μ*m. Sections that included the optic nerve were stained with Multiple Stain Solution (Polysciences, Inc, Warminster, PA, USA). For immunohistochemistry, eyes were fixed in 4% PFA for 2 hours at room temperature. The posterior segment was processed for whole mount immunostaining as has been previously described.^9, 18, 19, 29, 55^ For whole mount immunostaining, retinas were dissected free of the optic cup, blocked in 10% horse serum in 0.3% TritonX in 1 × PBS overnight at 4 °C, and incubated for three days at 4 °C in primary antibody. Primary antibodies included: rabbit anti-JUN (Abcam, 1:250, Cambridge, UK) and mouse *β*III tubulin (TUJ1, 1:000, Covance, San Diego, CA, USA). Whole retinas were washed, incubated at 4 °C in Alexafluor-conjugated secondary antibodies (Invitrogen, Carlsbad, CA, USA) for 48 h, washed, and mounted RGC side up in Fluorogel in TRIS buffer (Electron Microscopy Sciences). Quantification of TUJI+ and JUN+ cells was completed on eight × 40 fields per retina equally spaced 220*μ*m from the peripheral edge of the retina.^18, 19^ The cell-counter tool in ImageJ was used for quantification.

### Statistical analysis

For all experimental conditions, at least four retinas were analyzed for each genotype. During quantification of cell counts, the experimenters were masked to genotype and/or experimental cohort. Experiments comparing differences across two groups were analyzed using the unpaired Student’s *t*-test. Experiments comparing differences at a single time across more than two groups were analyzed using a one-way ANOVA followed by the Bonferroni *post hoc* test for group comparisons. Experiments comparing differences across more than one time point with more than two groups were analyzed using a two-way ANOVA followed by the Bonferroni *post hoc* test for group comparisons. Statistical significance was considered *P-*values<0.05.

## References

[bib1] Gordon MO, Beiser JA, Brandt JD, Heuer DK, Higginbotham EJ, Johnson CA et al. The Ocular Hypertension Treatment Study: baseline factors that predict the onset of primary open-angle glaucoma. Arch Ophthalmol 2002; 120: 714–720. 12049575 10.1001/archopht.120.6.714

[bib2] Nickells RW. The cell and molecular biology of glaucoma: mechanisms of retinal ganglion cell death. Invest Ophthalmol Vis Sci 2012; 53: 2476–2481. 22562845 10.1167/iovs.12-9483h PMC3990459

[bib3] Quigley HA, Hohman RM, Addicks EM, Massof RW, Green WR. Morphologic changes in the lamina cribrosa correlated with neural loss in open-angle glaucoma. Am J Ophthalmol 1983; 95: 673–691. 6846459 10.1016/0002-9394(83)90389-6

[bib4] Howell GR, Libby RT, Jakobs TC, Smith RS, Phalan FC, Barter JW et al. Axons of retinal ganglion cells are insulted in the optic nerve early in DBA/2J glaucoma. J Cell Biol 2007; 179: 1523–1537. 18158332 10.1083/jcb.200706181 PMC2373494

[bib5] Schlamp CL, Li Y, Dietz JA, Janssen KT, Nickells RW. Progressive ganglion cell loss and optic nerve degeneration in DBA/2J mice is variable and asymmetric. BMC Neurosci 2006; 7: 66. 17018142 10.1186/1471-2202-7-66 PMC1621073

[bib6] Jakobs TC, Libby RT, Ben Y, John SW, Masland RH. Retinal ganglion cell degeneration is topological but not cell type specific in DBA/2J mice. J Cell Biol 2005; 171: 313–325. 16247030 10.1083/jcb.200506099 PMC2171185

[bib7] Anderson DR, Hendrickson A. Effect of intraocular pressure on rapid axoplasmic transport in monkey optic nerve. Invest Ophthalmol 1974; 13: 771–783. 4137635

[bib8] Maes ME, Schlamp CL, Nickells RW. BAX to basics: How the BCL2 gene family controls the death of retinal ganglion cells. Prog Retin Eye Res 2017; 57: 1–25. 28064040 10.1016/j.preteyeres.2017.01.002 PMC5350025

[bib9] Libby RT, Li Y, Savinova OV, Barter J, Smith RS, Nickells RW et al. Susceptibility to neurodegeneration in a glaucoma is modified by Bax gene dosage. PLoS Genet 2005; 1: 17–26. 16103918 10.1371/journal.pgen.0010004 PMC1183523

[bib10] Isenmann S, Engel S, Gillardon F, Bahr M. Bax antisense oligonucleotides reduce axotomy-induced retinal ganglion cell death *in vivo* by reduction of Bax protein expression. Cell Death Differ 1999; 6: 673–682. 10453078 10.1038/sj.cdd.4400538

[bib11] Li Y, Schlamp CL, Poulsen KP, Nickells RW. Bax-dependent and independent pathways of retinal ganglion cell death induced by different damaging stimuli. Exp Eye Res 2000; 71: 209–213. 10930325 10.1006/exer.2000.0873

[bib12] Semaan SJ, Li Y, Nickells RW. A single nucleotide polymorphism in the Bax gene promoter affects transcription and influences retinal ganglion cell death. ASN Neuro 2010; 2: e00032. 20360947 10.1042/AN20100003 PMC2847828

[bib13] Lukas TJ, Wang AL, Yuan M, Neufeld AH. Early cellular signaling responses to axonal injury. Cell Commun Signal 2009; 7: 5. 19284657 10.1186/1478-811X-7-5 PMC2661080

[bib14] Yang J, Wu Z, Renier N, Simon DJ, Uryu K, Park DS et al. Pathological axonal death through a MAPK cascade that triggers a local energy deficit. Cell 2015; 160: 161–176. 25594179 10.1016/j.cell.2014.11.053 PMC4306654

[bib15] Abe N, Cavalli V. Nerve injury signaling. Curr Opin Neurobiol 2008; 18: 276–283. 18655834 10.1016/j.conb.2008.06.005 PMC2633416

[bib16] Geden MJ, Deshmukh M. Axon degeneration: context defines distinct pathways. Curr Opin Neurobiol 2016; 39: 108–115. 27197022 10.1016/j.conb.2016.05.002 PMC4987202

[bib17] Tezel G, Chauhan BC, LeBlanc RP, Wax MB. Immunohistochemical assessment of the glial mitogen-activated protein kinase activation in glaucoma. Invest Ophthalmol Vis Sci 2003; 44: 3025–3033. 12824248 10.1167/iovs.02-1136

[bib18] Fernandes KA, Harder JM, Fornarola LB, Freeman RS, Clark AF, Pang IH et al. JNK2 and JNK3 are major regulators of axonal injury-induced retinal ganglion cell death. Neurobiol Dis 2012; 46: 393–401. 22353563 10.1016/j.nbd.2012.02.003 PMC3323666

[bib19] Fernandes KA, Harder JM, Kim J, Libby RT. JUN regulates early transcriptional responses to axonal injury in retinal ganglion cells. Exp Eye Res 2013; 112: 106–117. 23648575 10.1016/j.exer.2013.04.021 PMC3700614

[bib20] Levkovitch-Verbin H, Quigley HA, Martin KR, Harizman N, Valenta DF, Pease ME et al. The transcription factor c-jun is activated in retinal ganglion cells in experimental rat glaucoma. Exp Eye Res 2005; 80: 663–670. 15862173 10.1016/j.exer.2004.11.016

[bib21] Isenmann S, Bahr M. Expression of c-Jun protein in degenerating retinal ganglion cells after optic nerve lesion in the rat. Exp Neurol 1997; 147: 28–36. 9294400 10.1006/exnr.1997.6585

[bib22] Munemasa Y, Ohtani-Kaneko R, Kitaoka Y, Kumai T, Kitaoka Y, Hayashi Y et al. Pro-apoptotic role of c-Jun in NMDA-induced neurotoxicity in the rat retina. J Neurosci Res 2006; 83: 907–918. 16477618 10.1002/jnr.20786

[bib23] Roth S, Shaikh AR, Hennelly MM, Li Q, Bindokas V, Graham CE. Mitogen-activated protein kinases and retinal ischemia. Invest Ophthalmol Vis Sci 2003; 44: 5383–5395. 14638742 10.1167/iovs.03-0451

[bib24] Gesslein B, Hakansson G, Carpio R, Gustafsson L, Perez MT, Malmsjo M. Mitogen-activated protein kinases in the porcine retinal arteries and neuroretina following retinal ischemia-reperfusion. Mol Vis 2010; 16: 392–407. 20300568 PMC2838742

[bib25] Pelzel HR, Schlamp CL, Nickells RW. Histone H4 deacetylation plays a critical role in early gene silencing during neuronal apoptosis. BMC Neurosci 2010; 11: 62. 20504333 10.1186/1471-2202-11-62 PMC2886060

[bib26] Kwong JM, Caprioli J. Expression of phosphorylated c-Jun N-terminal protein kinase (JNK) in experimental glaucoma in rats. Exp Eye Res 2006; 82: 576–582. 16197943 10.1016/j.exer.2005.08.017

[bib27] Tezel G, Yang X, Yang J, Wax MB. Role of tumor necrosis factor receptor-1 in the death of retinal ganglion cells following optic nerve crush injury in mice. Brain Res 2004; 996: 202–212. 14697498 10.1016/j.brainres.2003.10.029

[bib28] Raivich G, Behrens A. Role of the AP-1 transcription factor c-Jun in developing, adult and injured brain. Prog Neurobiol 2006; 78: 347–363. 16716487 10.1016/j.pneurobio.2006.03.006

[bib29] Harder JM, Libby RT. BBC3 (PUMA) regulates developmental apoptosis but not axonal injury induced death in the retina. Mol Neurodegener 2011; 6: 50. 21762490 10.1186/1750-1326-6-50 PMC3149592

[bib30] Howell GR, Soto I, Libby RT, John SW. Intrinsic axonal degeneration pathways are critical for glaucomatous damage. Exp Neurol 2013; 246: 54–61. 22285251 10.1016/j.expneurol.2012.01.014 PMC3831512

[bib31] Nickells RW, Howell GR, Soto I, John SW. Under pressure: cellular and molecular responses during glaucoma, a common neurodegeneration with axonopathy. Annu Rev Neurosci 2012; 35: 153–179. 22524788 10.1146/annurev.neuro.051508.135728

[bib32] Kerschensteiner M, Schwab ME, Lichtman JW, Misgeld T. *in vivo* imaging of axonal degeneration and regeneration in the injured spinal cord. Nat Med 2005; 11: 572–577. 15821747 10.1038/nm1229

[bib33] Raff MC, Whitmore AV, Finn JT. Axonal self-destruction and neurodegeneration. Science 2002; 296: 868–871. 11988563 10.1126/science.1068613

[bib34] Xiong X, Collins CA. A conditioning lesion protects axons from degeneration via the Wallenda/DLK MAP kinase signaling cascade. J Neurosci 2012; 32: 610–615. 22238096 10.1523/JNEUROSCI.3586-11.2012 PMC3280217

[bib35] Libby RT, Anderson MG, Pang IH, Robinson ZH, Savinova OV, Cosma IM et al. Inherited glaucoma in DBA/2J mice: pertinent disease features for studying the neurodegeneration. Vis Neurosci 2005; 22: 637–648. 16332275 10.1017/S0952523805225130

[bib36] John SW, Smith RS, Savinova OV, Hawes NL, Chang B, Turnbull D et al. Essential iris atrophy, pigment dispersion, and glaucoma in DBA/2J mice. Invest Ophthalmol Vis Sci 1998; 39: 951–962. 9579474

[bib37] Chang B, Smith RS, Hawes NL, Anderson MG, Zabaleta A, Savinova O et al. Interacting loci cause severe iris atrophy and glaucoma in DBA/2J mice. Nat Genet 1999; 21: 405–409. 10192392 10.1038/7741

[bib38] Libby RT, Gould DB, Anderson MG, John SW. Complex genetics of glaucoma susceptibility. Annu Rev Genomics Hum Genet 2005; 6: 15–44. 16124852 10.1146/annurev.genom.6.080604.162209

[bib39] Matsubara A, Nakazawa T, Husain D, Iliaki E, Connolly E, Michaud NA et al. Investigating the effect of ciliary body photodynamic therapy in a glaucoma mouse model. Invest Ophthalmol Vis Sci 2006; 47: 2498–2507. 16723462 10.1167/iovs.05-0959

[bib40] Anderson MG, Libby RT, Mao M, Cosma IM, Wilson LA, Smith RS et al. Genetic context determines susceptibility to intraocular pressure elevation in a mouse pigmentary glaucoma. BMC Biol 2006; 4: 20. 16827931 10.1186/1741-7007-4-20 PMC1543659

[bib41] Wong AA, Brown RE. A neurobehavioral analysis of the prevention of visual impairment in the DBA/2J mouse model of glaucoma. Invest Ophthalmol Vis Sci 2012; 53: 5956–5966. 22859742 10.1167/iovs.12-10020

[bib42] Schuettauf F, Quinto K, Naskar R, Zurakowski D. Effects of anti-glaucoma medications on ganglion cell survival: the DBA/2J mouse model. Vision Res 2002; 42: 2333–2337. 12350421 10.1016/s0042-6989(02)00188-8

[bib43] Behrens A, Sibilia M, David JP, Mohle-Steinlein U, Tronche F, Schutz G et al. Impaired postnatal hepatocyte proliferation and liver regeneration in mice lacking c-jun in the liver. EMBO J 2002; 21: 1782–1790. 11927562 10.1093/emboj/21.7.1782 PMC125360

[bib44] Furuta Y, Lagutin O, Hogan BL, Oliver GC. Retina- and ventral forebrain-specific Cre recombinase activity in transgenic mice. Genesis 2000; 26: 130–132. 10686607

[bib45] Templeton JP, Nassr M, Vazquez-Chona F, Freeman-Anderson NE, Orr WE, Williams RW et al. Differential response of C57BL/6J mouse and DBA/2J mouse to optic nerve crush. BMC Neurosci 2009; 10: 90. 19643015 10.1186/1471-2202-10-90 PMC2727955

[bib46] Li Y, Semaan SJ, Schlamp CL, Nickells RW. Dominant inheritance of retinal ganglion cell resistance to optic nerve crush in mice. BMC Neurosci 2007; 8: 19. 17338819 10.1186/1471-2202-8-19 PMC1831479

[bib47] Harder JM, Fernandes KA, Libby RT. The Bcl-2 family member BIM has multiple glaucoma-relevant functions in DBA/2J mice. Sci Rep 2012; 2: 530. 22833783 10.1038/srep00530 PMC3404412

[bib48] Wang S, Park S, Fei P, Sorenson CM. Bim is responsible for the inherent sensitivity of the developing retinal vasculature to hyperoxia. Dev Biol 2011; 349: 296–309. 21047504 10.1016/j.ydbio.2010.10.034 PMC3021136

[bib49] Howell GR, MacNicoll KH, Braine CE, Soto I, Macalinao DG, Sousa GL et al. Combinatorial targeting of early pathways profoundly inhibits neurodegeneration in a mouse model of glaucoma. Neurobiol Dis 2014; 71: 44–52. 25132557 10.1016/j.nbd.2014.07.016 PMC4319373

[bib50] Howell GR, Macalinao DG, Sousa GL, Walden M, Soto I, Kneeland SC et al. Molecular clustering identifies complement and endothelin induction as early events in a mouse model of glaucoma. J Clin Invest 2011; 121: 1429–1444. 21383504 10.1172/JCI44646 PMC3069778

[bib51] Whitmore AV, Libby RT, John SW. Glaucoma: thinking in new ways-a role for autonomous axonal self-destruction and other compartmentalised processes? Prog Retin Eye Res 2005; 24: 639–662. 15953750 10.1016/j.preteyeres.2005.04.004

[bib52] Adalbert R, Nogradi A, Szabo A, Coleman MP. The slow Wallerian degeneration gene *in vivo* protects motor axons but not their cell bodies after avulsion and neonatal axotomy. Eur J Neurosci 2006; 24: 2163–2168. 17074042 10.1111/j.1460-9568.2006.05103.x

[bib53] Lorber B, Tassoni A, Bull ND, Moschos MM, Martin KR. Retinal ganglion cell survival and axon regeneration in WldS transgenic rats after optic nerve crush and lens injury. BMC Neurosci 2012; 13: 56. 22672534 10.1186/1471-2202-13-56 PMC3404964

[bib54] Beirowski B, Babetto E, Coleman MP, Martin KR. The WldS gene delays axonal but not somatic degeneration in a rat glaucoma model. Eur J Neurosci 2008; 28: 1166–1179. 18783366 10.1111/j.1460-9568.2008.06426.x

[bib55] Fernandes KA, Harder JM, John SW, Shrager P, Libby RT. DLK-dependent signaling is important for somal but not axonal degeneration of retinal ganglion cells following axonal injury. Neurobiol Dis 2014; 69: 108–116. 24878510 10.1016/j.nbd.2014.05.015 PMC4099422

[bib56] Nickells RW. From ocular hypertension to ganglion cell death: a theoretical sequence of events leading to glaucoma. Can J Ophthalmol 2007; 42: 278–287. 17392853

[bib57] Wang AL, Yuan M, Neufeld AH. Degeneration of neuronal cell bodies following axonal injury in Wld(S) mice. J Neurosci Res 2006; 84: 1799–1807. 17022038 10.1002/jnr.21075

[bib58] Ju KR, Kim HS, Kim JH, Lee NY, Park CK. Retinal glial cell responses and Fas/FasL activation in rats with chronic ocular hypertension. Brain Res 2006; 1122: 209–221. 17045251 10.1016/j.brainres.2006.09.022

[bib59] Naskar R, Wissing M, Thanos S. Detection of early neuron degeneration and accompanying microglial responses in the retina of a rat model of glaucoma. Invest Ophthalmol Vis Sci 2002; 43: 2962–2968. 12202516

[bib60] Howell GR, Soto I, Zhu X, Ryan M, Macalinao DG, Sousa GL et al. Radiation treatment inhibits monocyte entry into the optic nerve head and prevents neuronal damage in a mouse model of glaucoma. J Clin Invest 2012; 122: 1246–1261. 22426214 10.1172/JCI61135 PMC3314470

[bib61] Lam TT, Kwong JM, Tso MO. Early glial responses after acute elevated intraocular pressure in rats. Invest Ophthalmol Vis Sci 2003; 44: 638–645. 12556393 10.1167/iovs.02-0255

[bib62] Wang JW, Chen SD, Zhang XL, Jonas JB. Retinal Microglia in Glaucoma. J Glaucoma 2016; 25: 459–465. 25646715 10.1097/IJG.0000000000000200

[bib63] Hernandez MR, Miao H, Lukas T. Astrocytes in glaucomatous optic neuropathy. Prog Brain Res 2008; 173: 353–373. 18929121 10.1016/S0079-6123(08)01125-4

[bib64] Hunt D, Hossain-Ibrahim K, Mason MR, Coffin RS, Lieberman AR, Winterbottom J et al. ATF3 upregulation in glia during Wallerian degeneration: differential expression in peripheral nerves and CNS white matter. BMC Neurosci 2004; 5: 9. 15113454 10.1186/1471-2202-5-9 PMC400733

[bib65] Guo Y, Johnson EC, Cepurna WO, Dyck JA, Doser T, Morrison JC. Early gene expression changes in the retinal ganglion cell layer of a rat glaucoma model. Invest Ophthalmol Vis Sci 2011; 52: 1460–1473. 21051717 10.1167/iovs.10-5930 PMC3101663

[bib66] Yang Z, Quigley HA, Pease ME, Yang Y, Qian J, Valenta D et al. Changes in gene expression in experimental glaucoma and optic nerve transection: the equilibrium between protective and detrimental mechanisms. Invest Ophthalmol Vis Sci 2007; 48: 5539–5548. 18055803 10.1167/iovs.07-0542

[bib67] Guo Y, Johnson E, Cepurna W, Jia L, Dyck J, Morrison JC. Does elevated intraocular pressure reduce retinal TRKB-mediated survival signaling in experimental glaucoma? Exp Eye Res 2009; 89: 921–933. 19682984 10.1016/j.exer.2009.08.003 PMC2783343

[bib68] Svensson C, Part K, Kunnis-Beres K, Kaldmae M, Fernaeus SZ, Land T. Pro-survival effects of JNK and p38 MAPK pathways in LPS-induced activation of BV-2 cells. Biochem Biophys Res Commun 2011; 406: 488–492. 21338578 10.1016/j.bbrc.2011.02.083

[bib69] Yu C, Minemoto Y, Zhang J, Liu J, Tang F, Bui TN et al. JNK suppresses apoptosis via phosphorylation of the proapoptotic Bcl-2 family protein BAD. Mol Cell 2004; 13: 329–340. 14967141 10.1016/s1097-2765(04)00028-0

[bib70] Granato M, Santarelli R, Lotti LV, Di Renzo L, Gonnella R, Garufi A et al. JNK and macroautophagy activation by bortezomib has a pro-survival effect in primary effusion lymphoma cells. PLoS One 2013; 8: e75965. 24086672 10.1371/journal.pone.0075965 PMC3784388

[bib71] Watkins TA, Wang B, Huntwork-Rodriguez S, Yang J, Jiang Z, Eastham-Anderson J et al. DLK initiates a transcriptional program that couples apoptotic and regenerative responses to axonal injury. Proc Natl Acad Sci USA 2013; 110: 4039–4044. 23431164 10.1073/pnas.1211074110 PMC3593899

[bib72] Welsbie DS, Yang Z, Ge Y, Mitchell KL, Zhou X, Martin SE et al. Functional genomic screening identifies dual leucine zipper kinase as a key mediator of retinal ganglion cell death. Proc Natl Acad Sci USA 2013; 110: 4045–4050. 23431148 10.1073/pnas.1211284110 PMC3593842

[bib73] Davis MI, Hunt JP, Herrgard S, Ciceri P, Wodicka LM, Pallares G et al. Comprehensive analysis of kinase inhibitor selectivity. Nat Biotechnol 2011; 29: 1046–1051. 22037378 10.1038/nbt.1990

[bib74] Karaman MW, Herrgard S, Treiber DK, Gallant P, Atteridge CE, Campbell BT et al. A quantitative analysis of kinase inhibitor selectivity. Nat Biotechnol 2008; 26: 127–132. 18183025 10.1038/nbt1358

[bib75] Shin JE, Miller BR, Babetto E, Cho Y, Sasaki Y, Qayum S et al. SCG10 is a JNK target in the axonal degeneration pathway. Proc Natl Acad Sci USA 2012; 109: E3696–E3705. 23188802 10.1073/pnas.1216204109 PMC3535671

[bib76] Miller BR, Press C, Daniels RW, Sasaki Y, Milbrandt J, DiAntonio A. A dual leucine kinase-dependent axon self-destruction program promotes Wallerian degeneration. Nat Neurosci 2009; 12: 387–389. 19287387 10.1038/nn.2290 PMC2696160

[bib77] Yang L, Li S, Miao L, Huang H, Liang F, Teng X et al. Rescue of glaucomatous neurodegeneration by differentially modulating neuronal endoplasmic reticulum stress molecules. J Neurosci 2016; 36: 5891–5903. 27225776 10.1523/JNEUROSCI.3709-15.2016 PMC4879204

[bib78] Babetto E, Beirowski B, Russler EV, Milbrandt J, DiAntonio A. The Phr1 ubiquitin ligase promotes injury-induced axon self-destruction. Cell Rep 2013; 3: 1422–1429. 23665224 10.1016/j.celrep.2013.04.013 PMC3671584

[bib79] Gerdts J, Sasaki Y, Vohra B, Marasa J, Milbrandt J. Image-based screening identifies novel roles for IkappaB kinase and glycogen synthase kinase 3 in axonal degeneration. J Biol Chem 2011; 286: 28011–28018. 21685387 10.1074/jbc.M111.250472 PMC3151046

[bib80] Anderson MG, Smith RS, Hawes NL, Zabaleta A, Chang B, Wiggs JL et al. Mutations in genes encoding melanosomal proteins cause pigmentary glaucoma in DBA/2J mice. Nat Genet 2002; 30: 81–85. 11743578 10.1038/ng794

